# Crystal structure of 1,2,3,4-di-*O*-methyl­ene-α-d-galacto­pyran­ose

**DOI:** 10.1107/S2056989015021854

**Published:** 2015-11-21

**Authors:** Ioannis Tiritiris, Stefan Tussetschläger, Willi Kantlehner

**Affiliations:** a Fakultät Chemie/Organische Chemie, Hochschule Aalen, Beethovenstrasse 1, D-73430 Aalen, Germany

**Keywords:** crystal structure, de­acetyl­ation, d-galactose, O—H⋯O hydrogen bonds, C—H⋯O hydrogen bonds

## Abstract

The title compound, C_8_H_12_O_6_, was synthesized by de­acetyl­ation of 6-acetyl-1,2,3,4-di-*O*-methyl­ene-α-d-galactose with sodium methoxide. The central part of the mol­ecule consists of a six-membered C_5_O pyran­ose ring with a twist-boat conformation. Both fused dioxolane rings adopt an envelope conformation with C and O atoms as the flap. In the crystal, O—H⋯O and C—H⋯O hydrogen bonds are present between adjacent mol­ecules, generating a three-dimensional network.

## Related literature   

For the synthesis of 6-acetyl-1,2,3,4-di-*O*-methyl­ene-α-d-galactose, see: Bok *et al.* (1952 ▸). For the crystal structures of the α- and β-anomers of d-galactose, see: Sheldrick (1976 ▸). For the crystal structure of 6-*O*-cyano­methyl-1,2:3,4-di-*O*-iso­propyl­idene-α-d-galactose, see: Langer *et al.* (2005 ▸). For the crystal structure of 6-[bis­(eth­oxy­carbon­yl)meth­yl]-6-de­oxy-1,2;3,4-di-*O*-iso­propyl­idene-d-galacto­pyran­ose, see: Doboszewski *et al.* (2010 ▸). For the crystal structure of 1,2,3,5-di-*O*-methyl­ene-α-d-xylo­furan­ose see: Tiritiris *et al.* (2015*a* ▸).
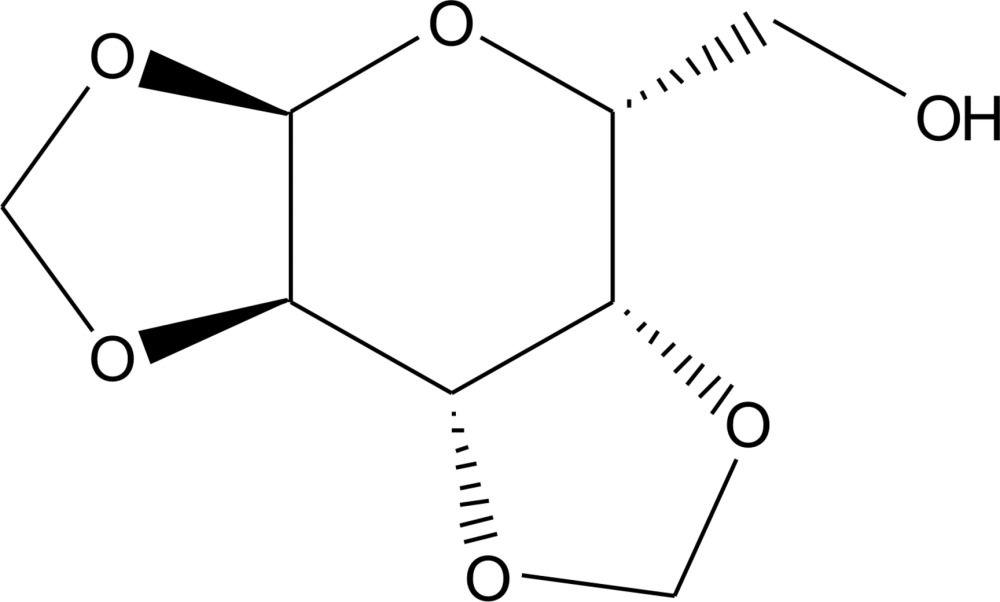


## Experimental   

### Crystal data  

C_8_H_12_O_6_
*M* *_r_* = 204.18
Orthorhombic, 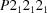

*a* = 6.4876 (6) Å
*b* = 6.6364 (5) Å
*c* = 20.1224 (16) Å
*V* = 866.36 (12) Å^3^
*Z* = 4
Mo *K*α radiation
μ = 0.14 mm^−1^
*T* = 100 K
0.43 × 0.32 × 0.04 mm

### Data collection  

Bruker Kappa APEXII DUO diffractometer
Absorption correction: multi-scan (Blessing, 1995 ▸) *T*_min_ = 0.705, *T*_max_ = 0.746
10453 measured reflections
2680 independent reflections
2464 reflections with *I* > 2σ(*I*)
*R*_int_ = 0.023
Standard reflections: 0

### Refinement  

*R*[*F* ^2^ > 2σ(*F* ^2^)] = 0.029
*wR*(*F* ^2^) = 0.075
*S* = 1.06
2680 reflections
131 parameters
H atoms treated by a mixture of independent and constrained refinement
Δρ_max_ = 0.31 e Å^−3^
Δρ_min_ = −0.18 e Å^−3^

### 

Data collection: *APEX2* (Bruker, 2008 ▸); cell refinement: *SAINT* (Bruker, 2008 ▸); data reduction: *SAINT*; program(s) used to solve structure: *SHELXS97* (Sheldrick, 2008 ▸); program(s) used to refine structure: *SHELXL2014* (Sheldrick, 2015 ▸); molecular graphics: *DIAMOND* (Brandenburg & Putz, 2005 ▸); software used to prepare material for publication: *SHELXL2014*.

## Supplementary Material

Crystal structure: contains datablock(s) I, global. DOI: 10.1107/S2056989015021854/zl2651sup1.cif

Structure factors: contains datablock(s) I. DOI: 10.1107/S2056989015021854/zl2651Isup2.hkl

Supporting information file. DOI: 10.1107/S2056989015021854/zl2651Isup3.cml

**Fig. 1.**
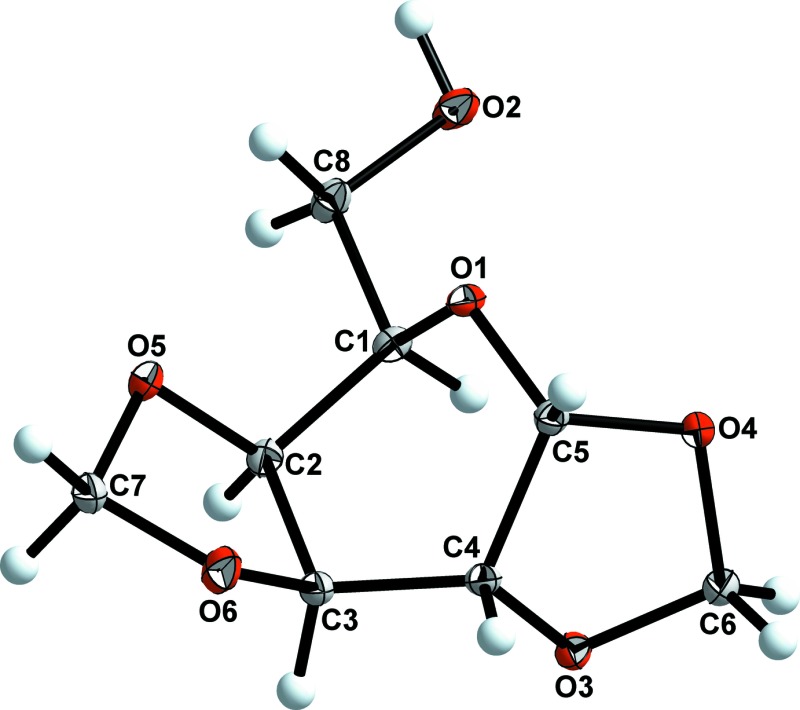
The structure of the title compound with displacement ellipsoids at the 50% probability level.

**Fig. 2.**
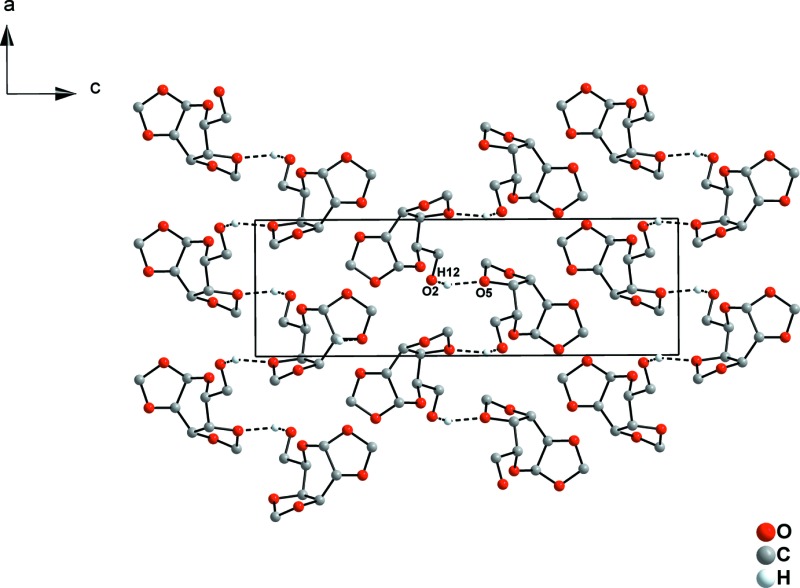
O—H···O hydrogen bonds (black dashed lines) between adjacent molecules in the crystal structure of the title compound (ac view).

**Fig. 3.**
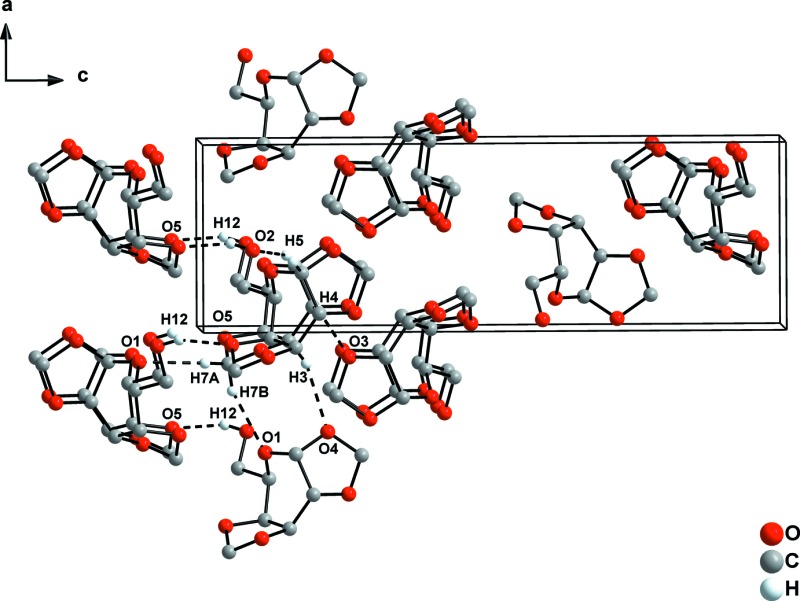
C—H···O and O—H···O hydrogen bonds (black dashed lines) between adjacent molecules in the crystal structure of the title compound (ac view).

. DOI: 10.1107/S2056989015021854/zl2651fig1.tif
The structure of the title compound with displacement ellipsoids at the 50% probability level.

ac . DOI: 10.1107/S2056989015021854/zl2651fig2.tif
O—H⋯O hydrogen bonds (black dashed lines) between adjacent mol­ecules in the crystal structure of the title compound (*ac* view).

ac . DOI: 10.1107/S2056989015021854/zl2651fig3.tif
C—H⋯O and O—H⋯O hydrogen bonds (black dashed lines) between adjacent mol­ecules in the crystal structure of the title compound (*ac* view).

CCDC reference: 1437272

Additional supporting information:  crystallographic information; 3D view; checkCIF report

## Figures and Tables

**Table 1:** Hydrogen-bond geometry (Å, °)

*D*—H⋯*A*	*D*—H	H⋯*A*	*D*⋯*A*	*D*—H⋯*A*
O2—H12⋯O5^i^	0.87 (3)	2.01 (3)	2.846 (2)	161
C3—H3⋯O4^ii^	1.00	2.49	3.447 (2)	160
C4—H4⋯O3^iii^	1.00	2.46	3.296 (2)	141
C5—H5⋯O2^iv^	1.00	2.45	3.405 (2)	160
C7—H7*A*⋯O1^v^	0.99	2.48	3.455 (2)	169
C7—H7*B*⋯O1^ii^	0.99	2.56	3.509 (2)	162

Symmetry codes: (i) 
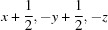
; (ii) 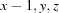; (iii) 
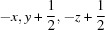
; (iv) 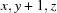; (v) 
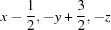
.

## supplementary crystallographic information

### S1. Comment

The synthesis of the protected sugar 1,2,3,4-di-*O*-methylene-α-*D*-galactopyranose has been well known for many years (Bok *et al.*, 1952). Its crystal structure, however, remained undetermined. According to the structure analysis of the title compound, which we would like to report now, the central part of the molecule consists of a six-membered C_5_O ring, which is made up from the carbon atoms C1–C5 and O1 (Fig. 1). The chiral carbon atoms C1/C2/C4/C5 of the pyranose part show *R*-configuration and C3 shows S-configuration, in agreement with the expected configurations for an α-*D*-galactose ring. Both fused dioxolane rings adopt an envelope conformation with the carbon atom C7 (ring I) and oxygen atom O3 (ring II) as the flap, respectively. The pyranose ring shows a twist-boat conformation. A similar conformation of the pyranose ring has been observed in 6-[bis(ethoxycarbonyl)methyl]-6-deoxy-1,2;3,4-di-*O*-isopropylidene-*D*-galactopyranose (Doboszewski *et al.*, 2010). The C–O and C–C and bond lengths in the molecule are comparable with the data from the crystal structure analysis of 1,2,3,5-di-*O*-methylene-α-*D*-xylofuranose (Tiritiris *et al.*, 2015*a*) and other related compounds [see, for example: 6-*O*-cyanomethyl-1,2:3,4-di-*O*-isopropylidene-α-*D*-galactose (Langer *et al.*, 2005), and the α- and β-anomers of *D*-galactose (Sheldrick, 1976)]. In the crystal structure, O—H···O hydrogen bonds between adjacent molecules are present [*d*(H···O) = 2.01 (3) Å] (Tab. 1), generating infinite one-dimensional chains with base vector [100] (Fig. 2). Taking additional C—H···O hydrogen bonds between adjacent molecules [*d*(H···O) = 2.45–2.56 Å] (Tab. 1) into account, a three-dimensional network is generated (Fig. 3).

### S2. Experimental

According to the literature (Bok *et al.*, 1952) a solution of 25 g (139 mmol) *D*-galactose in 20 ml water was mixed with 100 ml glacial acetic acid. 27.5 g (916 mmol) paraformaldehyde was then added at room temperature. Followed by dropwise addition of 12.5 ml concentrated sulfuric acid, the reaction mixture was heated to 373 K for one hour. After subsequent cooling to room temperature, 100 ml water was added to the mixture. The solution was extracted three times with chloroform and the combined extracts were washed with water and dried over sodium sulfate. After evaporation of the solvent, the crude product was destilled under reduced presure using a 20 cm *Vigreux* column. The fraction at 407 K (0.1 mbar) contained 4.28 g (13%) of 6-acetyl-1,2,3,4-di-*O*-methylene-α-*D*-galactose as the product. To a heated solution of 2.96 g (12 mmol) 6-acetyl-1,2,3,4-di-*O*-methylene-α-*D*-galactose (Bok *et al.*, 1952) in 25 ml me thanol, 50 mg of sodium methoxide was added. After subsequent cooling to room temperature, 50 ml water was added to the mixture. The solution was extracted two times with diethyl ether and the combined extracts were dried over sodium sulfate. After evaporation of the solvent, the crude product was distilled under reduced presure using a 20 cm *Vigreux* column. The fraction at 395 K (0.1 mbar) contained 1.6 g (66%) of the title compound, which crystallized spontaneously after several days at room temperature, forming colorless single crystals suitable for X-ray analysis.

### S3. Refinement

The O-bound H atom was located in a difference Fourier map and was refined freely [O2—H12 = 0.87 (3) Å]. The title compound crystallizes in the non-centrosymmetric space group *P2_1_2_1_2_1_*; however, in the absence of significant anomalous scattering effects, the Flack parameter is essentially meaningless and the absolute configuration was chosen based on known stereocenters unchanged during synthesis. The H atoms in CH_2_ and CH groups were placed in calculated positions with d(C—H) = 0.99 Å and d(C—H) = 1.00 Å and refined using a riding model, with *U*_eq_(H) set to 1.2 *U*_eq_(C).

### Crystal data

**Table:**

C_8_H_12_O_6_	*D*_x_ = 1.565 Mg m^−^^3^
*M**_r_* = 204.18	Mo *K*α radiation, λ = 0.71073 Å
Orthorhombic, *P*2_1_2_1_2_1_	Cell parameters from 2468 reflections
*a* = 6.4876 (6) Å	θ = 2.0–30.7°
*b* = 6.6364 (5) Å	µ = 0.14 mm^−^^1^
*c* = 20.1224 (16) Å	*T* = 100 K
*V* = 866.36 (12) Å^3^	Plate, colorless
*Z* = 4	0.43 × 0.32 × 0.04 mm
*F*(000) = 432	

### Data collection

**Table:**

Bruker Kappa APEXII DUO diffractometer	2680 independent reflections
Radiation source: fine-focus sealed tube	2464 reflections with *I* > 2σ(*I*)
Triumph monochromator	*R*_int_ = 0.023
φ scans, and ω scans	θ_max_ = 30.7°, θ_min_ = 2.0°
Absorption correction: multi-scan (Blessing, 1995)	*h* = −9→6
*T*_min_ = 0.705, *T*_max_ = 0.746	*k* = −9→8
10453 measured reflections	*l* = −28→25

### Refinement

**Table:**

Refinement on *F*^2^	Primary atom site location: structure-invariant direct methods
Least-squares matrix: full	Secondary atom site location: difference Fourier map
*R*[*F*^2^ > 2σ(*F*^2^)] = 0.029	Hydrogen site location: inferred from neighbouring sites
*wR*(*F*^2^) = 0.075	H atoms treated by a mixture of independent and constrained refinement
*S* = 1.06	*w* = 1/[σ^2^(*F*_o_^2^) + (0.044*P*)^2^ + 0.0725*P*] where *P* = (*F*_o_^2^ + 2*F*_c_^2^)/3
2680 reflections	(Δ/σ)_max_ < 0.001
131 parameters	Δρ_max_ = 0.31 e Å^−^^3^
0 restraints	Δρ_min_ = −0.18 e Å^−^^3^

### Special details

**Table:**

Geometry. All e.s.d.'s (except the e.s.d. in the dihedral angle between two l.s. planes) are estimated using the full covariance matrix. The cell e.s.d.'s are taken into account individually in the estimation of e.s.d.'s in distances, angles and torsion angles; correlations between e.s.d.'s in cell parameters are only used when they are defined by crystal symmetry. An approximate (isotropic) treatment of cell e.s.d.'s is used for estimating e.s.d.'s involving l.s. planes.
Refinement. Refinement of *F*^2^ against ALL reflections. The weighted *R*-factor *wR* and goodness of fit *S* are based on *F*^2^, conventional *R*-factors *R* are based on *F*, with *F* set to zero for negative *F*^2^. The threshold expression of *F*^2^ > σ(*F*^2^) is used only for calculating *R*-factors(gt) *etc*. and is not relevant to the choice of reflections for refinement. *R*-factors based on *F*^2^ are statistically about twice as large as those based on *F*, and *R*- factors based on ALL data will be even larger.

### Fractional atomic coordinates and isotropic or equivalent isotropic displacement parameters (Å^2^)

**Table:**

	*x*	*y*	*z*	*U*_iso_*/*U*_eq_	
O1	0.34564 (16)	0.56520 (14)	0.10994 (5)	0.0131 (2)	
C1	0.2020 (2)	0.4035 (2)	0.11836 (7)	0.0131 (3)	
H1	0.2178	0.3452	0.1639	0.016*	
O2	0.44408 (19)	0.15283 (16)	0.08328 (5)	0.0213 (2)	
H12	0.479 (4)	0.093 (4)	0.0465 (13)	0.046 (7)*	
C2	−0.0156 (2)	0.4835 (2)	0.11004 (6)	0.0144 (3)	
H2	−0.1186	0.3778	0.1224	0.017*	
O3	0.12624 (15)	0.64670 (16)	0.25197 (5)	0.0161 (2)	
C3	−0.0551 (2)	0.6797 (2)	0.15012 (6)	0.0151 (3)	
H3	−0.1768	0.6608	0.1800	0.018*	
O4	0.45725 (16)	0.63594 (16)	0.21656 (5)	0.0173 (2)	
C4	0.1274 (2)	0.7537 (2)	0.19020 (6)	0.0134 (3)	
H4	0.1135	0.9015	0.1987	0.016*	
O5	−0.04486 (17)	0.54598 (15)	0.04200 (5)	0.0173 (2)	
C5	0.3420 (2)	0.70826 (19)	0.16104 (7)	0.0129 (2)	
H5	0.4051	0.8365	0.1447	0.016*	
O6	−0.10172 (18)	0.82816 (16)	0.10055 (5)	0.0209 (2)	
C6	0.3323 (2)	0.6556 (2)	0.27434 (7)	0.0162 (3)	
H6A	0.3608	0.5448	0.3060	0.019*	
H6B	0.3596	0.7857	0.2968	0.019*	
C7	−0.1757 (2)	0.7162 (2)	0.04575 (7)	0.0193 (3)	
H7A	−0.1678	0.7967	0.0044	0.023*	
H7B	−0.3206	0.6746	0.0530	0.023*	
C8	0.2518 (3)	0.2439 (2)	0.06717 (7)	0.0179 (3)	
H8A	0.1419	0.1405	0.0666	0.021*	
H8B	0.2594	0.3057	0.0225	0.021*	

### Atomic displacement parameters (Å^2^)

**Table:**

	*U*^11^	*U*^22^	*U*^33^	*U*^12^	*U*^13^	*U*^23^
O1	0.0137 (5)	0.0135 (4)	0.0122 (4)	−0.0019 (4)	0.0031 (4)	−0.0021 (3)
C1	0.0159 (6)	0.0117 (5)	0.0117 (6)	−0.0017 (5)	0.0011 (5)	0.0002 (4)
O2	0.0284 (6)	0.0197 (5)	0.0156 (5)	0.0096 (5)	−0.0014 (5)	−0.0029 (4)
C2	0.0142 (6)	0.0171 (6)	0.0118 (6)	−0.0039 (5)	0.0002 (5)	0.0014 (5)
O3	0.0139 (5)	0.0230 (5)	0.0115 (4)	−0.0016 (4)	0.0002 (4)	0.0030 (4)
C3	0.0129 (6)	0.0206 (6)	0.0119 (6)	0.0025 (5)	0.0008 (5)	0.0006 (5)
O4	0.0127 (5)	0.0262 (5)	0.0131 (4)	0.0037 (4)	−0.0017 (4)	−0.0034 (4)
C4	0.0137 (6)	0.0151 (5)	0.0113 (6)	0.0026 (5)	0.0015 (5)	0.0003 (5)
O5	0.0174 (5)	0.0238 (5)	0.0105 (4)	0.0015 (4)	−0.0018 (4)	0.0003 (4)
C5	0.0134 (6)	0.0125 (5)	0.0129 (6)	−0.0007 (5)	0.0000 (5)	−0.0014 (4)
O6	0.0249 (6)	0.0220 (5)	0.0158 (5)	0.0072 (4)	−0.0051 (4)	0.0008 (4)
C6	0.0155 (6)	0.0203 (6)	0.0128 (6)	−0.0005 (5)	−0.0005 (5)	−0.0008 (5)
C7	0.0141 (7)	0.0295 (7)	0.0144 (6)	0.0036 (6)	−0.0020 (5)	0.0015 (5)
C8	0.0237 (8)	0.0145 (6)	0.0155 (7)	0.0020 (6)	−0.0025 (5)	−0.0022 (5)

### Geometric parameters (Å, º)

**Table:**

O1—C5	1.3997 (16)	C3—H3	1.0000
O1—C1	1.4313 (16)	O4—C6	1.4233 (17)
C1—C8	1.5123 (19)	O4—C5	1.4275 (17)
C1—C2	1.518 (2)	C4—C5	1.5404 (19)
C1—H1	1.0000	C4—H4	1.0000
O2—C8	1.4237 (19)	O5—C7	1.4152 (18)
O2—H12	0.87 (3)	C5—H5	1.0000
C2—O5	1.4430 (16)	O6—C7	1.4136 (18)
C2—C3	1.553 (2)	C6—H6A	0.9900
C2—H2	1.0000	C6—H6B	0.9900
O3—C6	1.4120 (17)	C7—H7A	0.9900
O3—C4	1.4313 (16)	C7—H7B	0.9900
C3—O6	1.4342 (17)	C8—H8A	0.9900
C3—C4	1.5146 (19)	C8—H8B	0.9900
			
C5—O1—C1	114.24 (10)	C5—C4—H4	109.8
O1—C1—C8	107.79 (11)	C7—O5—C2	104.94 (10)
O1—C1—C2	109.29 (10)	O1—C5—O4	109.75 (10)
C8—C1—C2	111.62 (11)	O1—C5—C4	115.33 (11)
O1—C1—H1	109.4	O4—C5—C4	103.95 (11)
C8—C1—H1	109.4	O1—C5—H5	109.2
C2—C1—H1	109.4	O4—C5—H5	109.2
C8—O2—H12	103.1 (18)	C4—C5—H5	109.2
O5—C2—C1	109.12 (11)	C7—O6—C3	104.66 (11)
O5—C2—C3	103.29 (11)	O3—C6—O4	105.95 (10)
C1—C2—C3	112.93 (11)	O3—C6—H6A	110.5
O5—C2—H2	110.4	O4—C6—H6A	110.5
C1—C2—H2	110.4	O3—C6—H6B	110.5
C3—C2—H2	110.4	O4—C6—H6B	110.5
C6—O3—C4	104.54 (10)	H6A—C6—H6B	108.7
O6—C3—C4	108.21 (12)	O6—C7—O5	104.91 (11)
O6—C3—C2	104.46 (10)	O6—C7—H7A	110.8
C4—C3—C2	114.78 (12)	O5—C7—H7A	110.8
O6—C3—H3	109.7	O6—C7—H7B	110.8
C4—C3—H3	109.7	O5—C7—H7B	110.8
C2—C3—H3	109.7	H7A—C7—H7B	108.8
C6—O4—C5	108.07 (11)	O2—C8—C1	109.23 (11)
O3—C4—C3	107.31 (11)	O2—C8—H8A	109.8
O3—C4—C5	103.80 (11)	C1—C8—H8A	109.8
C3—C4—C5	116.12 (11)	O2—C8—H8B	109.8
O3—C4—H4	109.8	C1—C8—H8B	109.8
C3—C4—H4	109.8	H8A—C8—H8B	108.3
			
C5—O1—C1—C8	−169.46 (11)	C3—C2—O5—C7	−24.54 (13)
C5—O1—C1—C2	69.05 (13)	C1—O1—C5—O4	80.45 (13)
O1—C1—C2—O5	66.87 (13)	C1—O1—C5—C4	−36.51 (15)
C8—C1—C2—O5	−52.27 (14)	C6—O4—C5—O1	−129.34 (12)
O1—C1—C2—C3	−47.39 (14)	C6—O4—C5—C4	−5.45 (13)
C8—C1—C2—C3	−166.53 (11)	O3—C4—C5—O1	103.14 (12)
O5—C2—C3—O6	0.05 (14)	C3—C4—C5—O1	−14.36 (17)
C1—C2—C3—O6	117.79 (12)	O3—C4—C5—O4	−17.05 (13)
O5—C2—C3—C4	−118.27 (12)	C3—C4—C5—O4	−134.55 (12)
C1—C2—C3—C4	−0.54 (16)	C4—C3—O6—C7	147.17 (12)
C6—O3—C4—C3	156.74 (11)	C2—C3—O6—C7	24.45 (14)
C6—O3—C4—C5	33.27 (13)	C4—O3—C6—O4	−37.75 (14)
O6—C3—C4—O3	159.69 (11)	C5—O4—C6—O3	26.67 (14)
C2—C3—C4—O3	−84.12 (13)	C3—O6—C7—O5	−41.25 (14)
O6—C3—C4—C5	−84.77 (14)	C2—O5—C7—O6	41.43 (14)
C2—C3—C4—C5	31.42 (16)	O1—C1—C8—O2	68.75 (14)
C1—C2—O5—C7	−144.91 (11)	C2—C1—C8—O2	−171.22 (11)

### Hydrogen-bond geometry (Å, º)

**Table:**

*D*—H···*A*	*D*—H	H···*A*	*D*···*A*	*D*—H···*A*
O2—H12···O5^i^	0.87 (3)	2.01 (3)	2.846 (2)	161
C3—H3···O4^ii^	1.00	2.49	3.447 (2)	160
C4—H4···O3^iii^	1.00	2.46	3.296 (2)	141
C5—H5···O2^iv^	1.00	2.45	3.405 (2)	160
C7—H7*A*···O1^v^	0.99	2.48	3.455 (2)	169
C7—H7*B*···O1^ii^	0.99	2.56	3.509 (2)	162

Symmetry codes: (i) *x*+1/2, −*y*+1/2, −*z*; (ii) *x*−1, *y*, *z*; (iii) −*x*, *y*+1/2, −*z*+1/2; (iv) *x*, *y*+1, *z*; (v) *x*−1/2, −*y*+3/2, −*z*.
