# Supplementary figures and images for: Crystal structure of 1,2,3,4-di-O-methyl­ene-α-d-galacto­pyran­ose
Source: Acta Crystallogr E Crystallogr Commun. 2015 Nov 21;71(Pt 12):o961–2. doi: 10.1107/S2056989015021854 (PMC4719923; doi:10.1107/S2056989015021854)

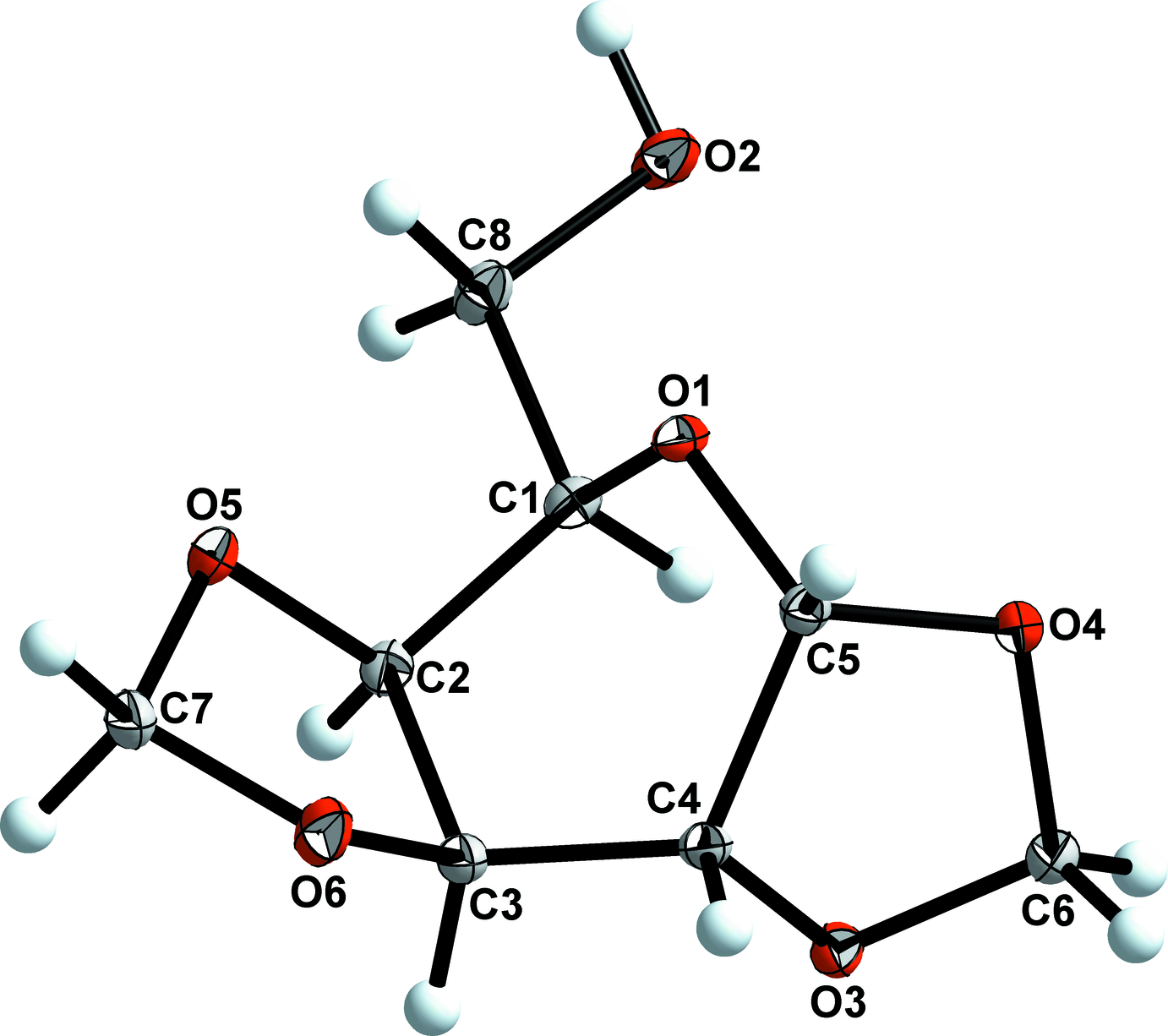

Supplement: Supplementary file 4 [file e-71-0o961-fig1.tif]

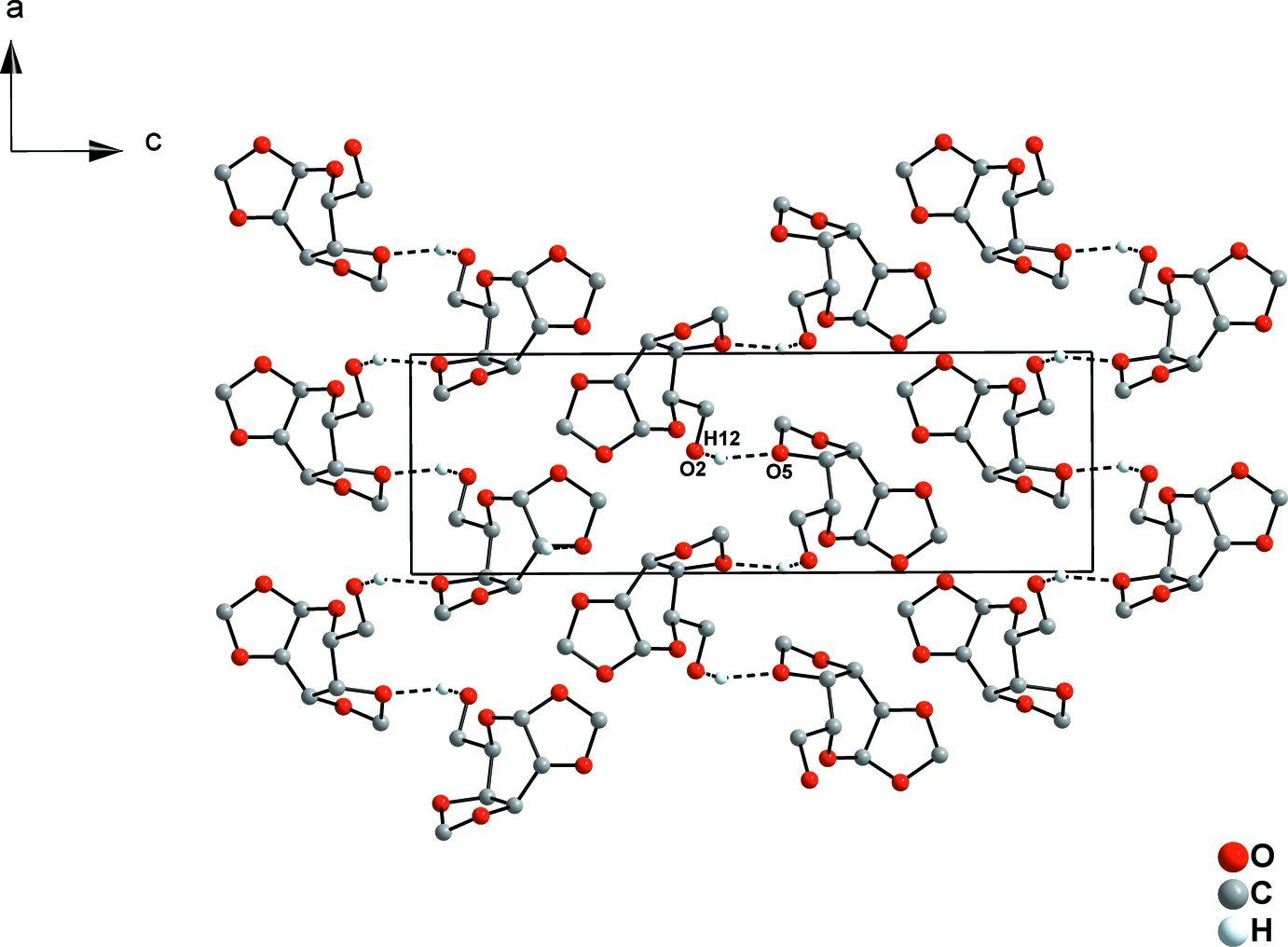

Supplement: Supplementary file 5 [file e-71-0o961-fig2.tif]

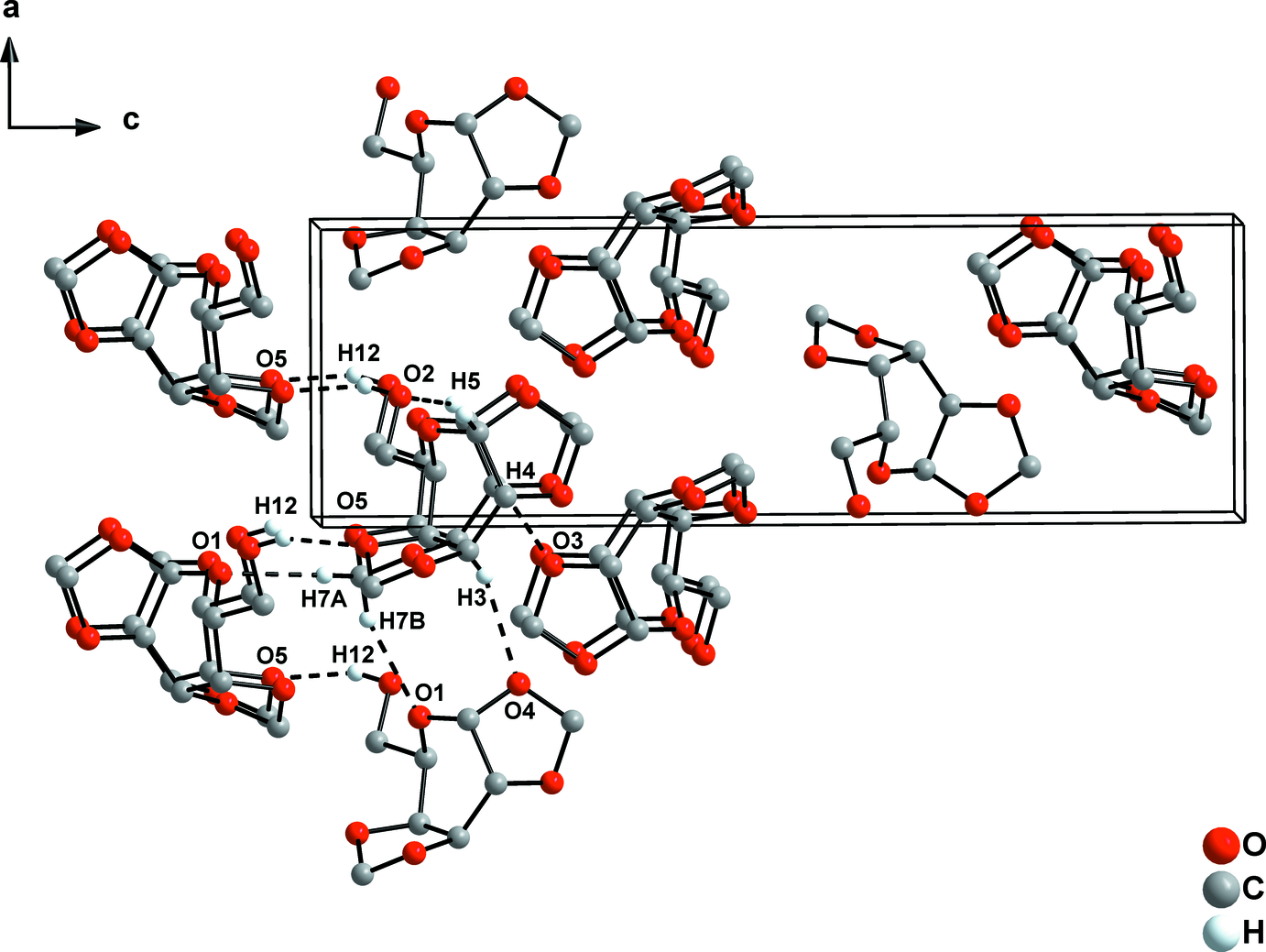

Supplement: Supplementary file 6 [file e-71-0o961-fig3.tif]
